# Silk Fibroin-Based Nanoparticles for Drug Delivery

**DOI:** 10.3390/ijms16034880

**Published:** 2015-03-04

**Authors:** Zheng Zhao, Yi Li, Mao-Bin Xie

**Affiliations:** 1 State Key Lab of Advanced Technology for Materials Synthesis and Processing, Wuhan University of Technology, Wuhan 430070, China; E-Mail: zhengzhao@whut.edu.cn; 2 Institute of Textiles and Clothing, the Hong Kong Polytechnic University, Hong Kong 999077, China; E-Mail: maobin.xie@connect.polyu.hk; 3 Biomedical Materials and Engineering Research Center of Hubei Province, Wuhan University of Technology, Wuhan 430070, China

**Keywords:** silk fibroin, nanoparticles, preparation methods, drug delivery

## Abstract

Silk fibroin (SF) is a protein-based biomacromolecule with excellent biocompatibility, biodegradability and low immunogenicity. The development of SF-based nanoparticles for drug delivery have received considerable attention due to high binding capacity for various drugs, controlled drug release properties and mild preparation conditions. By adjusting the particle size, the chemical structure and properties, the modified or recombinant SF-based nanoparticles can be designed to improve the therapeutic efficiency of drugs encapsulated into these nanoparticles. Therefore, they can be used to deliver small molecule drugs (e.g., anti-cancer drugs), protein and growth factor drugs, gene drugs, *etc.* This paper reviews recent progress on SF-based nanoparticles, including chemical structure, properties, and preparation methods. In addition, the applications of SF-based nanoparticles as carriers for therapeutic drugs are also reviewed.

## 1. Introduction

A drug delivery system consists of a drug carrier in which the active drug is dissolved, dispersed, or encapsulated, or onto which the active ingredient is adsorbed or attached [1]. Drug carrier materials play a significant role in the delivery of drug. These carriers can be processed into different drug-controlled release systems, such as nanoparticles, microspheres, microcapsules, pills, emulsions and so on.

Among them, nanoparticles have attracted much attention for their ability to be used as an effective carrier in promoting drug efficacy. Nanoparticles as drug carriers were first developed around the 1970s by Birrenbach and Speiser [2]. They were initially colloidal particulate systems with sizes ranging from 1~1000 nm, demonstrating unique characteristics because of their “size effect”. Nanopaticles may protect a drug from degradation, enhance biological stability, drug absorption into a selected tissue, bioavailability, retention time, intracellular penetration, and reduces patient expenses and risks of toxicity [3,4]. Furthermore, the desired drug release pattern biodistribution can be achieved by modulating the surface properties, composition and milieu [5].

Over the past few decades, many effective nanoparticle drug delivery systems have been developed. These nanoparticles generally can be prepared using various kinds of materials, such as liposomes, ceramics, carbon, metal, polymers, micelles, and dendrimers [6,7,8,9]. Especially, biodegradable polymer nanoparticles have been commonly used as drug delivery systems because of excellent biocompatibility, better encapsulation and controlled drug release properties. Various polymeric materials have been utilized as a drug delivery matrix, including the synthetic biodegradable polymers such as poly (lactic acid) (PLA), poly (ε-caprolactone) (PCL), and poly (glycolic acid) (PGA), and natural polymers such as polysaccharides, including cellulose, chitosan, hyaluronic acid, alginate, dextran, and starch, as well as proteins which contain collagen, gelatin, elastin, albumin, and silk fibroin [10].

There is growing interest in developing protein-based nanoparticle drug delivery systems due to their unique functionalities. Proteins-based carriers are biodegradable, non-antigenic, and possess excellent biocompatibility. Besides, proteins exhibit various functional groups and can trigger a biological response to cells. Especially, the surface of protein nanoparticles can be modified by covalent attachment of drugs and ligands to enhance therapeutic efficiency [11].

Silk Fibrin (SF) is a protein-based biomacromolecule. It has been extensively used in the biomedical fields as a biomaterial in the form of films, three-dimensional scaffolds, hydrogels, electrospun fibers, and spheres. Especially, its biodegradability, excellent biocompatibility, improvement of cell adhesion and proliferation, chemical modification potential and cross-linking possibility make SF-based nanoparticles a promising drug delivery system. This review paper will focus on SF-based nanoparticles used as a carrier for drug delivery. The chemical structure and properties of SF, preparation techniques of SF-based nanoparticles, and their applications as carriers for therapeutic drugs are discussed.

## 2. Chemical Structure and Properties of Silk Fibroin

Silk fibroin (SF) is a natural polymer spun by a variety of species including silkworms and spiders. The most well characterized silks are the dragline silk from the spider *Nephila clavipes* and the domesticated silkworm *Bombyx mori*. SF is a natural protein polymer that has been approved as a biomaterial by the US Food and Drug Administration (FDA). In contrast to the established supply chain available for silkworm silk, the commercial production of spider silks has been restricted owing to the more aggressive nature of spiders and the more complex and smaller quantities of silk mixtures generated in orb webs [12,13].

### 2.1. Silkworm (Bombyx mori) Silk Fibroin

SF is a protein-based biomacromolecule with bulky repetitive modular hydrophobic domains, which are interrupted by small hydrophilic groups. The primary structure of *Bombyx mori* SF is mainly composed of glycine (Gly) (43%), alanine (Ala) (30%) and serine (Ser) (12%) [14]. SF is a heterodimeric protein with a heavy (H) chain (~325 kDa) and a light (L) chain (~25 kDa) connected by a single disulfide bond at cys-172 of the L-chain and cys c-20 (twentieth residue from *C* terminus) of the H chain [15,16]. Also, a 25 kDa silk glycoprotein, P25 associated with disulfide-linked heavy and light chains by noncovalent interaction [17]. The chains of SF also contain amino acids with bulky and polar side chains, in particular tyrosine, valine, and acidic amino acids [18].

The H-chain of SF contains alternating hydrophobic and hydrophilic blocks similar to those seen in amphiphilic block co-polymers. It is hydrophobic and provides crystalline like features to the silk thread [19]. The hydrophobic domains of H chains contain Gly-X repeats, with X being Alanine (Ala), Serine (Ser), Threonine (Thr) and Valine (Val) and can form anti-parallel β-sheets and result in the stability and mechanical properties of the fiber. The hydrophilic links between these hydrophobic domains is non-repetitive and very short compared to the size of the hydrophobic repeats [20]. It consists of bulky and polar side chains and forms the amorphous part of the secondary structure. The chain conformation in amorphous blocks is random coil, which gives elasticity to silk [12,21]. The L-chain is hydrophilic in nature and relatively elastic. P25 protein could play a significant role in maintaining the integrity of the complex. The molar ratios of H-chain:L-chain:P25 are 6:6:1 [22,23].

### 2.2. Spider (Nephilia clavipes) Silk Fibroin

Unlike silk derived from *Bombyx mori*, spider silks do not have a sericin coating and may be used in natural fibre form or processed via formation of a spidroin solution. The most commonly studied spider silk is dragline silk from the spider *Nephila clavipes* [24]. Spider silk elicits almost no immunological response and has potential applications in the biomedical fields as a biomaterial for sutures, growth matrices, drug carrier and so on [25].

Dragline silk is produced in the major ampullate gland and is primarily comprised of two different proteins, major ampullate spidroin 1 (MaSp1) and major ampullate spidroin 2 (MaSp2) [26]. A single MaSp1 module usually consists of a hydrophobic polyalanine block and several hydrophilic GGX (where X is typically tyrosine, leucine or glutamine) motifs. In modules of MaSp2 the GGX motif is replaced by GPGXX [21,27]. The multiple repeats of hydrophobic polyalanine blocks (present in both proteins) are cross-linked and form crystalline β-sheets domains in silk proteins stabilized by hydrogen bonds and thus contribute to the high tensile strength of silk fibres. The crystalline β-sheets domains are separated by less organized hydrophilic blocks [28]. The blocks of GGX found in MaSp1 presumably form 3_10_-helices, and the blocks of GPGXX found only in MaSp2 form β-turn spirals imparting elasticity/flexibility to the proteins [29].

## 3. Preparation Methods of Silk Fibroin-Based Nanoparticles

There are several preparation methods available for the preparation of SF-based nanoparticles, such as desolvation, salting out, mechanical comminution, electrospraying, supercritical fluid technology and so on. Table 1 indicates the preparation methods of SF-based nanoparticles. Each method has pros and cons, so that selection of an appropriate method is important in formation of SF-based nanoaprticles for drug delivery applications. The fabrication of SF nanoparticles remains a challenging area that needs further exploration. The high molecular weight and protein nature of SF make the preparation of nanoparticles difficult to control. Moreover, SF tends to self-assemble into fibers or gels upon exposure to heat, salt, pH change and high shear.

**Table 1:** The preparation methods of SF-based nanoparticles.

Preparation Methods	Advantages	Disadvantages	Particle Size
Desolvation	Comparatively mild conditions; Small particle size; Simplicity of operation.	Easy to aggregate; low drug load; Organic solvent residue.	35~125 nm [30]; 150~170 nm [17]; 0.2~1.5 mm [31]; 980 nm [32]
Salting out	Low cost; high yield; Simplicity and safe operation; Avoids use of toxic solvents; Easy to maintain activity of protein.	Salting out agents residue.	486~1200 nm [33]
Supercritical fluid technologies	Low and no organic solvent residue; Comparatively high drug load; Controllable particle size.	High cost and High requirement (high pressure) for equipment; Complicated operation; Needs post treatment to induce insolubility of SF.	52.5~102.3 nm [34]
Electrospraying	Particle with high purity and excellent monodispersity; Controllable particle size; Simplicity of operation.	Needs post treatment to induce insolubility of SF.	80 nm [35]; 59~75 nm [36]
Mechanical comminution	Simplicity of operation; Easy to scale up.	Particle with big size and wide size distribution; The impurities and any grinding aids need be removed.	200 nm [37]; 700 nm [38]; ~200 nm [39]; ~200 nm [40]
Microemulsion method	Controllable particle size.	Residual surfactant and organic solvent may result in toxic problems.	167~169 nm [41]
Electric fields	Mild operation conditions; No use of organic solvent.	Particle with big size.	200 nm~3 μm [42]
Capillary-microdot technique	Simplicity of operation.	Organic solvent residue.	less than 100 nm [43]
PVA blend film method	Mild operation conditions; Easy and safe to manipulate; Time and energy efficient; No use of organic solvent.	PVA residue.	500 nm~2 mm [44]

### 3.1. Desolvation

**Figure 1:**
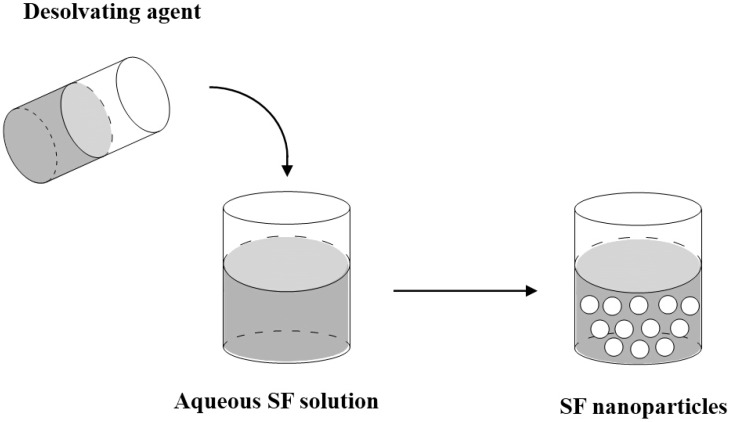
Schematic diagram of the desolvation method for preparing silk fibroin (SF) nanoparticles.

The desolvation/coacervation process is the most commonly used method to prepare protein-based nanoparticles due to comparatively mild conditions. The desolvation (simple coacervation) process reduces the solubility of the protein leading to phase separation. The addition of desolvating agent leads to conformation changes in protein structure resulting in coacervation or precipitation of the protein [11,45,46]. Figure 1 shows the schematic diagram of the desolvation method for preparing SF nanoparticles. In brief, the protein is initially dissolved in a solvent and then gradually extracted into a non-solvent phase. By phase separation, a phase with a colloidal component/coacervate and a second phase with a solvent/non-solvent mixture are formed. In this process, the solvent must be miscible with the non-solvent. A stable particle size is reached after an initial process period so that further desolvation (addition of non-solvent) solely leads to an increased particle yield. As the coacervation process is faster and more efficient at conditions of zero net charge (isoelectric point of the protein), the pH of the protein solution is of major importance and can be adjusted towards the desired conditions regarding particle size and process yield.

Zhang *et al.* [45] reported the preparation of the SF nanoparticles by mixing the aqueous SF and water miscible protonic organic solvents (methanol, ethanol, propanol and isopropanol) or polar aprotonic organic solvents (tetrahydrofurnan and acetone). In this process, the regenerated SF molecules were instantly converted from Silk I into Silk II. When the aqueous SF was introduced rapidly into acetone, the water-insoluble SF nanoparticles with β-structure and a range of 35~125 nm in diameter could be obtained.

Kundu *et al.* [17] prepared SF nanoparticles by desolvation technique using dimethyl sulfoxide (DMSO) as desolvating agent. The fabrication process consisted of the following steps namely, protein isolation, desolvation, centrifugation, purification, sonication, filtration and lyophilization. The nanoparticles were stable, spherical, negatively charged, 150~170 nm in diameter and exhibited mostly Silk II (β-sheet) structure and did not impose any overt toxicity.

Cao *et al.* [46] reported that SF microspheres, with predictable and controllable sizes ranging from 0.2 to 1.5 mm, were prepared by adding a small amount of ethanol into regenerated SF solution and quenching the mixture below the freezing point. The particle size and size distribution could be controlled by the conditions of preparation, such as the amount of ethanol added and the freezing temperature.

In order to prevent the agglomeration of silk particles, Shi *et al.* [32] prepared SF particles with an average diameter of 980 nm by employing polyvinyl alcohol (PVA) as an emulsifier in the process of particle formation. Briefly, SF solution was mixed with ethanol completely then vortex for 10 s. The PVA solution was then added to the silk/ethanol mixture and vortexed for another 10 s. The ternary solution was finally placed into a freezer for 24 h to form SF particles.

### 3.2. Salting out

A simple approach for preparation of protein-based nanoparticles is the salting out of a protein solution to form protein coacervates. Proteins have hydrophilic and hydrophobic parts. Hydrophobic parts can interact with the water molecules and allow proteins to form hydrogen bonds with the surrounding water molecules. With the increase of the salt concentration, the salt ions attract some of the water molecules, resulting in the removal of the water barrier between protein molecules and the increase of the protein-protein interactions. Therefore, the protein molecules aggregate together by forming hydrophobic interactions with each other and precipitate from the solution.

**Figure 2:**
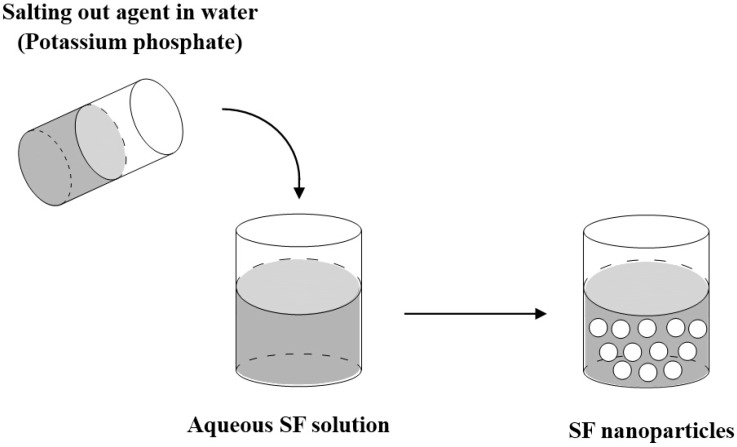
Schematic diagram of the salting out method for preparing SF nanoparticles.

Lammel *et al.* [33] reported the formation of SF nanoparticles with an average diameter of 486~1200 nm in an all-aqueous process by salting out with potassium phosphate (>0.75 m). Figure 2 shows the schematic diagram of the salting out method for preparing SF nanoparticles. Briefly, the silk fibroin solution was mixed with potassium phosphate. The resulting particles were then stored in the refrigerator for 2 h and could be collected by centrifugation. When using 1.25 m potassium phosphate (pH 8), increasing the concentration of SF can result in larger particles. Below pH 5 the particles aggregated into non-dispersible clusters. The small molecule model drugs, such as alcian blue, rhodamine B, and crystal violet, were loaded into the SF particles by simple absorption based on electrostatic interactions. The drug-loaded SF nanparticles showed a controlled release property, which depends on charge–charge interactions between the compounds and the SF.

### 3.3. Supercritical Fluid Technologies

Recently, very attractive new supercritical fluid (SCF) technologies have been established as useful alternatives to conventional methods for preparation of particles, avoiding the disadvantages of conventional techniques [47,48]. Supercritical fluids (SCFs) are substances at temperature and pressure conditions above their respective critical values (Pc; Tc). SCFs have unique thermo-physical properties and can penetrate substances like a gas and dissolve substances like a liquid. Among all the possible SCFs, supercritical CO_2_ (scCO_2_) is the most widely used and has been shown to have great potential in the field of micronization of materials because of its favorable critical conditions (Tc = 31.1 °C, Pc = 7.38 MPa), non-toxicity, non-flammability, and low costs from the viewpoint of pharmaceutical, nutraceutical and food applications [49,50].

**Figure 3:**
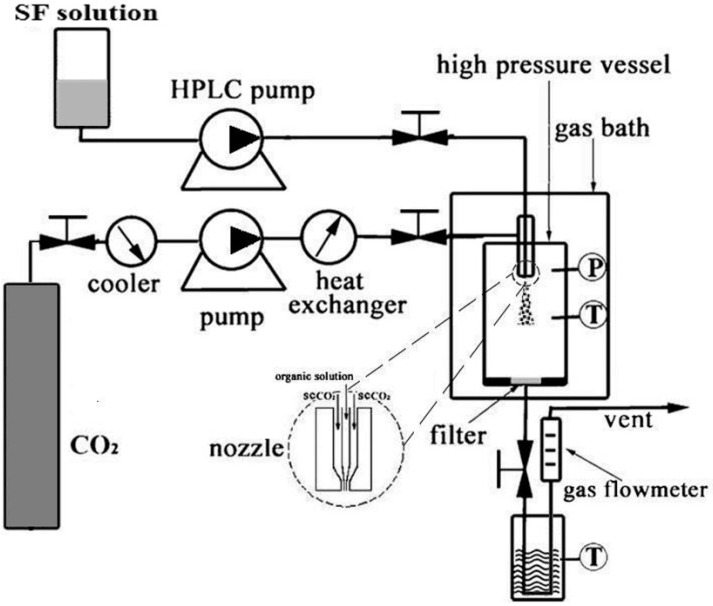
Schematic diagram of the SEDS process for preparing SF nanoparticles. Adapted with permission from [34]. Copyright 2013 American Chemical Society.

So far, the most common techniques for particle formation using scCO_2_ include the rapid expansion of supercritical solutions (RESS), particles from gas-saturated solutions or suspensions (PGSS), and gas or supercritical fluid antisolvent (GAS or SAS) [51,52,53]. In particular, solution-enhanced dispersion by supercritical fluids (SEDS), a modified SAS process, has been widely used to prepare micro or nanoparticles. Figure 3 shows the schematic diagram of the SEDS process for preparing SF nanoparticles. In this process, the solution containing solute and supercritical CO_2_ (scCO_2_) are atomized via a specially designed coaxial nozzle to obtain droplets with small size and enhance mixing to increase mass transfer rates. In this process, a nozzle with two coaxial passages allows the introduction of scCO_2_ and a solution into the high-pressure vessel where pressure and temperature are controlled [54]. When the solution contacts the scCO_2_, the high velocity of the scCO_2_ breaks up the solution into very small droplets and enhances mass transfer and mutual diffusion between SCFs and the droplets instantaneously, resulting in phase separation and supersaturation of the polymer solution, thus leading to nucleation and precipitation of the polymer particle [55]. In the SEDS process, the scCO_2_ acts as an anti-solvent. In addition, scCO_2_ is used as a “dispersing agent” to improve mass transfer between SCFs and the droplets. Therefore, very small particles can be produced. In addition, the particle size distribution and morphology of the polymer can be controlled by adjusting the parameters of the SEDS process, including the concentration of solute, flow rate of solution, temperature, and pressure of scCO_2_.

Zhao *et al.* [34] prepared SF nanoparticles with a particle size of about 50 nm via solution-enhanced dispersion by scCO_2_ (SEDS) for the first time successfully. The influence of process parameters on particle size and SF nanoparticles formation mechanism were investigated. The results indicated that precipitation temperature, concentration and flow rate of SF solution have a positive effect, while precipitation pressure has a negative effect. The nanoparticle formation mechanism was elucidated with the formation and growth of SF nuclei in the gaseous miscible phase evolved from initial droplets generated by the liquid-liquid phase split.

To utilize SF nanoparticles prepared by the SEDS process as drug carrier, Zhao *et al.* [10] fabricated the indomethacin (IDMC) loaded SF nanoparticles by the SEDS process. The results suggested SF nanoparticles exhibited excellent biocompatibility and time and concentration-dependent cellular uptake properties. An IDMC loading experiment suggested that the drug load (DL) and encapsulation efficiency (EE) of IDMC-SF nanoparticles were about 19.86% and 6.11% respectively. After the ethanol treatment, DL and EE of IDMC-SF nanoparticles decreased to 2.05% and 10.23%. An *in vitro* drug release experiment indicated that the accumulative release of IDMC from IDMC-SF nanoparticles was 61.15% after 6 h and reached 87% after 24 h. The drug release then reached a plateau, and only 5% more of the drug was released over the next 24 h. Obviously there is no burst effect and the drug is released in a stable way. In a word, the SF nanoparticles fabricated by the SEDS process are a potential drug carrier to be used in the biomedical field.

### 3.4. Electrospraying

**Figure 4:**
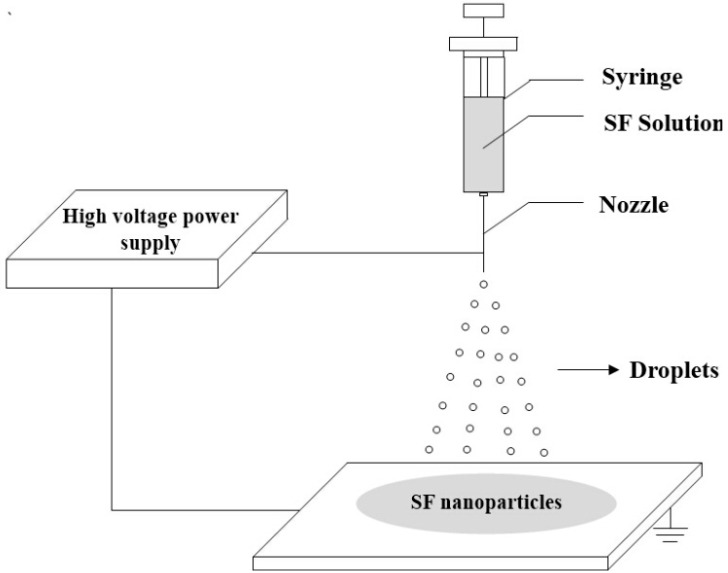
Schematic diagram of the electrospraying method for preparing SF nanoparticles.

Electrospraying (electrohydrodynamic spraying) is a method of liquid atomization by means of electrical forces and is an emerging method for the rapid and high throughput production of nanoparticles. Figure 4 shows the schematic diagram of the electrospraying method for preparing SF nanoparticles. In electrospraying, the liquid ﬂowing out of a capillary nozzle, which is maintained at high electric potential, is forced by the electric field to be dispersed into small droplets [56,57].

Gholami *et al.* [35] prepared SF nanoparticles with a uniform spherical shape and an average particle size as low as 80 nm by the electrospraying technique. Increasing the concentration of SF solutions, feed rate and needle-collector distance increased the average particle size of the SF nanoparticles. Increasing voltage decreased the particle size up to 20 kV, but with higher voltages (25 and 30 kV) the average particle size increases. The resulting SF nanoparticles exhibited a β-sheet structure, similar to fibroin filaments but with a lower crystallinity index. No functional group change occured in the process of electrospraying.

Qu *et al.* [36] fabricated SF nanoparticles with diameters ranging from 59 to 75 nm using high-voltage electrospray technology. Moreover, *cis*-dichlorodiamminoplatinum (CDDP) was incorporated into SF nanoparticles through metal-polymer coordination bond exchange. This study provided not only a novel method for preparing CDDP loaded SF nanoparticles but also a new delivery system for clinical therapeutic drugs against cancer.

### 3.5. Mechanical Comminution

**Figure 5:**
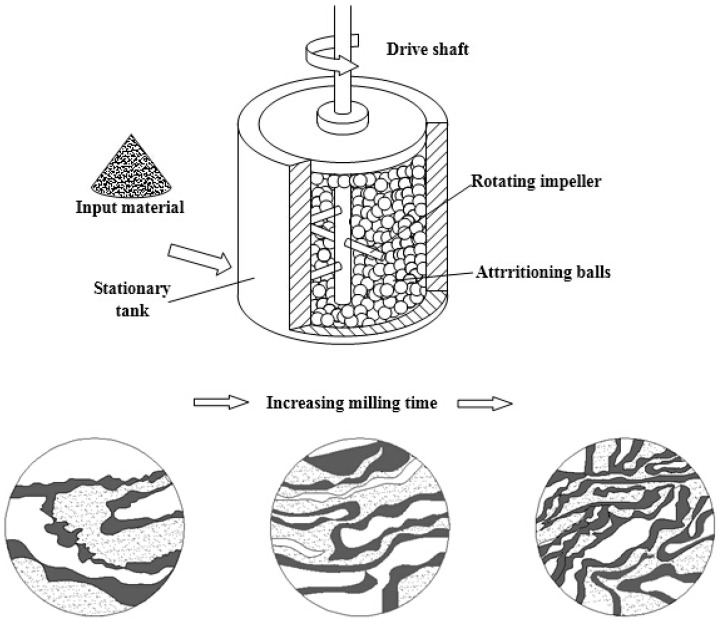
Schematic diagram of the mechanical comminution method for preparing particles.

Comminution is the reduction of solid materials from one average particle size to a smaller average particle size, by crushing, grinding, milling, *etc.* The method generally involves high energy dry/wet milling, with the addition of milling aids, and typically use milling times from several hours up to many days [58,59,60]. Figure 5 shows the schematic diagram of the mechanical comminution method for preparing particles. The method is easy to operate and scale up. However, the method still suffers from difficulties in ensuring that all the particles are milled properly. Long milling time also will result in more milling impurities. Moreover, the particle size distribution is wide. In addition, the impurities and any grinding aids used in the processing need be removed [60].

Rajkhowa *et al.* [37] prepared SF nanoparticles with a volume-based median particle size (d(0.5)) of around 200 nm by rotary and ball milling. In brief, degummed silk fibres were chopped into short snippets and then pulverised using rotary and planetary ball milling. Reduction in fibre strength via harsh degumming increased silk fragmentation rate, but also increased aggregation of SF particles. Water was helpful in the performance of ball milling. Rajkhowa *et al.* fabricated ultrafine silk powder with a volume based particle size d(0.5) of around 700 nm through attritor and jet milling [38]. The procedure include chopping of degummed silk, wet attritor milling, spray drying and air jet milling in chronological sequence. Unlike rotating container in a planetary ball mill used in the previous study [37], in the attritor, the balls are stirred inside a stationary container. Compared to the case for ball milling, this allows more irregular movement and spin to the media, resulting in a higher shear force and more frequent particle/media collision.

Kazemimostaghim *et al.* [39] fabricated SF nanoparticles by a combination of attritor and bead milling processes. Firstly, the silk fibres were degummed by alkaline hydrolysis. Then SF nanoparticles of volume median particle size d(0.5) of 7 μm in diameter was manufactured using attritor milling and subsequently reduced to ~200 nm with narrow particle size distribution by bead milling. Particle size was controlled by adjusting the pH and milling time. However, high pH may cause chemical damage to silk. To overcome the problem of alkali degradation above, Kazemimostaghim *et al.* [40] prepared SF nanoparticles using a bead milling method assisted by the biocompatible surfactant, Tween 80. The surfactant can be used to assist milling and prevent aggregation instead of utilizing repelling charges at high pH in the milling of submicron particles. In brief, silk particles with a volume median particle size (d(0.5)) of ~7 μm were obtained as a precursor, by attritor milling of silk snippets. The precursor particles were then bead milled using 0.5-mm beads and Tween 80. SF nanoparticles with d(0.5) of ~200 nm and narrow particle size distribution were fabricated by empolying 30% Tween 80 on the weight of powder.

### 3.6. Microemulsion Method

A microemulsion is a thermodynamically stable dispersion of two immiscible liquids (water and oil) with the aid of surfactant [61]. Small droplets of one liquid are stabilized in the other liquid by surfactant molecules accumulated at the oil-water interface. Microemulsions are generally classified into two types: water-in-oil (w/o), oil-in-water (o/w) and water-in-sc-CO_2_ (w/sc-CO_2_). In the w/o microemulsions, the aqueous phase forms nanometer-size droplets in a continuous hydrocarbon based continuous phase, and is normally located towards the oil apex of a water/oil/surfactant triangular phase diagram. In this region, the thermodynamically driven surfactant self-assembly generates aggregates known as reverse or inverted micelles. Spherical reverse micelles can minimise surface [62,63]. Adding a solvent like ethanol to the microemulsion, allows extraction of the precipitate by filtering or centrifuging the mixture. The main advantage of this method is better control on particle size by adjusting the nature and amount of surfactant and cosurfactant, the oil phase or the reacting conditions.

**Figure 6:**
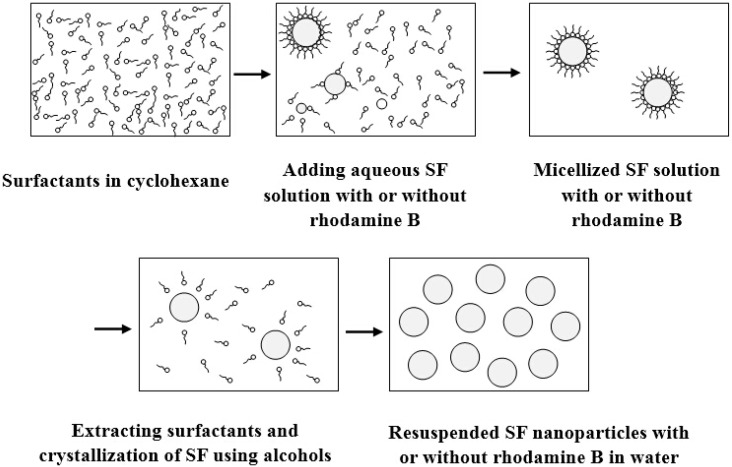
Schematic diagram of the microemulsion method for preparing SF nanoparticles. Adapted with permission from [41]. Copyright 2008 Springer.

Myung *et al.* [41] reported the preparation of SF nanoparticles prepared via a w/o microemulsion method. Figure 6 shows the schematic diagram of microemulsion method for preparing SF nanoparticles. In this procedure, Triton X-100 is employed as a surfactant. Firstly, the SF aqueous solution was added into the mixture of Triton X-100 cyclohexane under stirring. A mixture of methanol and ethanol was then added to remove surfactant, break the microemulsion and recover the particles. In addition, by mixing a color dye (rhodamine B) solution with the aqueous SF solution, fluorescent dye-encapsulated SF nanoparticles were obtained. The SF nanoparticles with/without fluorescent dye had an average size of about 167~169 nm. The observed stability of the fluorescent molecules in the SF nanoparticles indicated that these nanoparticles have a potential application in the fields of molecular imaging and bioassays.

### 3.7. Electric Fields

Leisk *et al.* reported an electrically mediated hydrogel (e-gel) from SF. The e-gel samples that were freeze-dried at −80 °C exhibited extended and spherical, micellar, micrometer-scale structures [64]. Lu Q *et al.* [65] found the formation of the SF nanoparticles with sizes of tens of nanometers was a critical step in the formation of e-gels. Under an electric field the nanoparticles aggregated to form nano- or microspheres on the positive electrodes owing to screening of the negative surface charge, which could otherwise prevent intermolecular self-assembly of SF in neutral solution.

**Figure 7:**
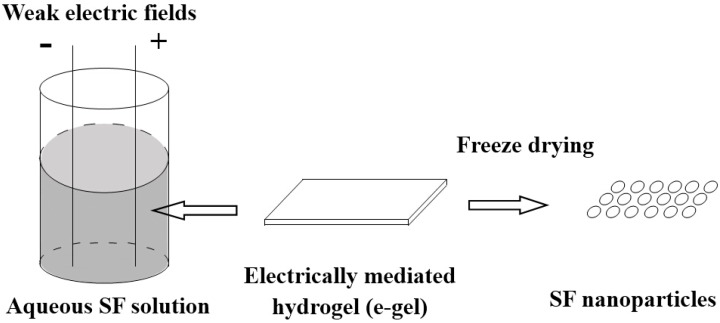
Schematic diagram of the electric fields method for preparing SF nanoparticles.

Based on the studies above, Huang *et al.* [42] prepared a SF gel system (e-gel) under weak electric fields containing SF microspheres with a diameter from about 200 nm to 3 μm. Figure 7 shows the schematic diagram of the electric fields method for preparing SF nanoparticles. Briefly, five groups of the SF solution was incubated for 6, 12, 24, 48 and 72 h at 70 °C respectively. Then electrodes were immersed in an aqueous solution of SF (0.8 wt %) and 25 V d.c. was applied over a 3 min period to a pair of conductive electrodes. Within seconds of the application of the voltage, a visible gel formed at the positive electrode. After washes using ddH_2_O, SF gels were placed into liquid nitrogen. Finally, the SF microspheres were formed by freeze drying. After heat treatment at 70 °C for 6 h, SF nanoparticles with a 300 nm in diameter were formed. After heat treatment at 70 °C for 6~24 h, some SF nanoparticles with a 500 nm in diameter formed. When increasing heat treatment to 48~72 h, SF microspheres with a diameter of 2~3 μm can be produced. Therefore, the size of the microspheres was controlled from about 200 nm to 3 μm by changing the incubation time at 70 °C. By adding bovine serum albumin labeled with fluorescein isothiocyanate (FITC-BSA) into SF solution, the SF microspheres containing FITC-BSA can be obtained. Considering the mild preparation conditions, the method for preparing SF drug-loaded systems has a potential application to load proteins and gene medicines with negative charge.

### 3.8. Capillary-Microdot Technique

**Figure 8:**
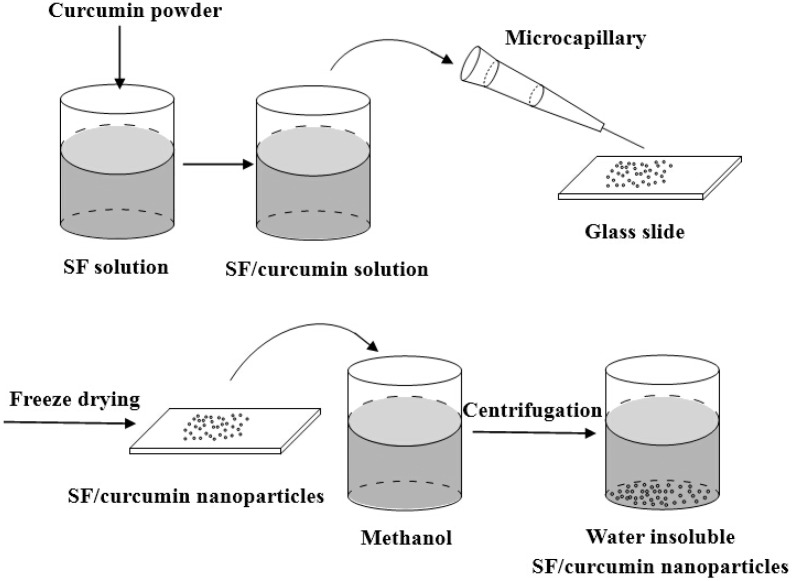
Schematic diagram of the capillary-microdot technique for preparing SF nanoparticles.

Gupta *et al.* [43] prepared SF-encapsulated curcumin nanoparticles less than 100 nm in size using the devised capillary-microdot technique. Figure 8 shows the schematic diagram of the capillary-microdot technique for preparing SF nanoparticles. In brief, curcumin was added into SF solution to form drug suspension. Then the suspension was dispensed on glass slides via a microcapillary. The slides were then frozen overnight and lyophilized. The resulting dry dots containing SF-encapsulated curcumin nanoparticles were scraped off the slides and were crystallized by methanol treatment. The nanoparticles collected by centrifugation was rinsed with phosphate-buffered saline (PBS) and were suspended in PBS for further analysis.

### 3.9. PVA Blend Film Method

**Figure 9:**
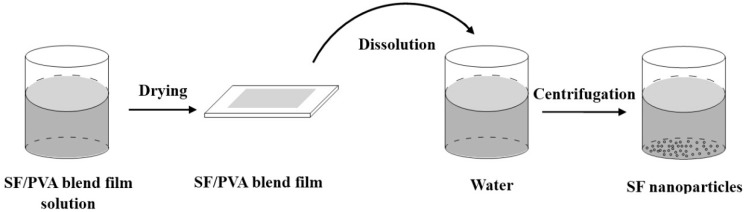
Schematic diagram of the PVA blend film method for preparing SF nanoparticles.

Wang *et al.* reported the formation of SF particles with controllable particle size (500 nm~2 mm) and shape using PVA as a continuous phase to separate SF solution into micro- and nanoparticles in SF/PVA blend films at a weight ratio from 1/1 to 1/4 [44]. The process was based on phase separation between SF and polyvinyl alcohol (PVA). Figure 9 shows the schematic diagram of the PVA blend film method for preparing SF nanoparticles. In brief, the SF/PVA blend solution was dried into a film firstly. Then, water-insoluble SF particles could be fabricated by film dissolution in water and subsequent centrifugation to remove PVA. The process was environmentally-friendly because the process only employed the water and the PVA, an FDA-approved substance. By adjusting the concentration of SF and PVA or employing ultrasonication on the blend solution, the SF particles with different particle size could be prepared. Drug can be loaded into SF particles by mixing model drugs in the original SF solution. These SF particles have potential as drug carriers in the field of biomedical applications.

## 4. The Applications of Silk Fibroin-Based Nanoparticles for Drug Delivery

A desirable drug carrier should be biodegradable, biocompatible, mechanically durable and could be prepared under mild conditions. In addition, the drug should be able to be released in a controlled manner [24]. Silk fibroin (SF) nanoparticles have been investigated as an ideal drug carrier candidate for a nanoparticle drug delivery system [22,66]. Especially, the SF exhibits several active amino groups and tyrosine residues that can be utilized for the conjugation of drugs, diagnostic agents, targeting ligands, and for surface modification for various biomedical applications [67]. Studies have shown that SF-based nanoparticles can carry various drugs, including small molecule drugs, protein drugs, and gene drugs, *etc.*

### 4.1. Small Molecule Drug Delivery

For small drug delivery from SF-based nanoparticles, significant research has focused on the delivery of anti-cancer drugs for cancer treatment. Most current anticancer agents are subjected to undesirable biodistribution, systemic toxicity and adverse side effects [68]. In order to treat tumors effectively, anti-cancer drugs should be delivered into the desired tumor tissues through many obstacles in the body with minimal loss of their therapeutic efficiency in the blood circulation. Once arriving in the area of tumor tissue, the anticancer drug can selectively kill targeted tumor cells without impairing normal cells by passive and active targeting [69,70]. Besides, the drugs should be released in a controlled manner in order to have the desired therapeutic effect.

Recently, the development of anti-cancer drug-loaded SF nanoparticles has shown significant potential for cancer treatment. Especially the incorporation of the anti-cancer drugs such as paclitaxel (PTX), doxorubicin (DOX), floxuridine, methotrexate, curcumin, emodin and *cis*-dichlorodiamminoplatinum into SF nanoparticles has gained much interest [71,72,73,74,75,76,77,78,79,80].

Chen *et al.* [71] prepared paclitaxel (PTX)-loaded silk fibroin (SF) nanoparticles ranging from 270 to 520 nm by addition of PTX-ethanol solution into regenerated SF solution under gentle stirring. The release time of PTX-SF nanoparticles can be as long as two weeks when the drug loading is about 3.0%. By a similar method, Wu *et al.* also prepared the PTX-SF nanoparticles with a diameter of 130 nm. PTX kept its pharmacological activity when incorporating into PTX-SF nanoparticles. The *in vivo* antitumor studies of PTX-SF nanoparticles on gastric cancer nude mice exnograft model indicated that that locoregional delivery of PTX-SF nanoparticles demonstrated superior antitumor efficacy by delaying tumor growth and reducing tumor weights compared with systemic administration [72].

Yu *et al.* [73] reported the formation of hydrophilic anti-cancer drug floxuridine-loaded SF nanoparticles with a particle size of 200~500 nm by a similar method. The maximum drug loading was about 6.8% and the release time of floxuridine was more than two days. The floxuridine-loaded SF nanoparticles possessed the similar curative effect to kill or inhibit Hela cells to the free floxuridine. After 24 h of incubation, floxuridine loaded SF nanoparticles inhibited more than 80% of Hela cells. These results together suggest that SF-based anti-cancer drug nanocarriers have great potential for lymphatic chemotherapy in clinical applications.

*Cis*-dichlorodiamminoplatinum (CDDP)-loaded SF nanoparticles with a particle size of about 59 nm were successfully fabricated by electrospray [49]. Cisplatin was released in a sustained way for more than 15 days. Moreover, the CDDP-loaded SF nanoparticles were internalized by A549 lung cancer cells and showed sustained inhibitory effect on tumor cells, but less toxicity to normal cells.

To enhance the therapeutic efficiency of emodin-loaded liposomes (ELP), Gobin *et al.* [74] prepared an SF-coated, emodin-loaded liposomes (SF-ELP). The SF coating restricted the abrupt swelling of liposomes and decreased the release rate of emodin. Besides, owing to the interaction of the SF molecular with the pericellular coating of the keloid fibroblasts, the addition of the SF coating also enhanced adhesive targeting to the keloids fibroblasts. Based on this study above, Cheema *et al.* [75] examined and compared the efficacy, adhesive targeting, and drug specificity of emodin delivered via SF-ELP *versus* ELP, against breast cancer cells that over-express the Her2/*neu* proto-oncogene. This investigation indicated that SF-mediated delivery of liposomal emodin showed higher efficacy in breast cancer cells. While the targeting is associated with the specificity of emodin for Her2/*neu* over-expressing cells, the SF coatings of the liposomes provide an enhanced delivery system to these cells by increasing the uptake/retention of emodin.

In order to adjust the properties of the SF nanoparticle drug delivery system such as the retention, efficacy, and bioavailability of curcumin, SF-chitosan nanoparticles were fabricated. The introduction of chitosan in the nanoparticle formulation of SF resulted in an increase of its hydrophilic character since chitosan is a water-carrying glucosamine molecule. Curcumin is a hydrophobic drug and hence the presence of chitosan with SF may have resulted in reducing the entrapment efficiency of curcumin. The size of the nanoparticles that showed high curcumin entrapment and efficacy towards breast cancer cells was less than 100 nm [64]. Nanoparticles of curcumin encapsulated with pure SF showed the highest curcumin entrapment, release, intracellular uptake, and efficacy towards breast cancer cells as compared to curcumin-loaded SF-chitosan nanoparticles. Therefore SF-based nanoparticles could be used as an anti-cancer drug carrier for the treatment of cancer and many other diseases.

The SF also has been used to improve the hydrophobic drugs encapsulation of other polymer such as albumin. The SF-albumin nanoparticles were prepared by desolvation method. Then the methotrexate was loaded on the SF-albumin nanoparticles by absorption. Increasing the content of the SF improved encapsulation efficiency of the SF-albumin nanoparticles and increased the release rate of hydrophobic methotrexate. Silk ﬁbroin contains hydrophobic amino acids and can form strong electrostatic interactions between the carboxyl groups of the SF and the amino groups of albumin. This helps prevent leakage of hydrophobic drugs from albumin, thereby improving encapsulation, drug retention and release rate. These nanoparticles exhibited a controlled drug release property. About 5% of the drug gets released after 12 days. Besides, these nanoparticles are easily internalized by the cells, reside within perinuclear spaces and act as carriers for delivery of the model drug methotrexate [76].

Although there have been some studies about the use of the SF nanoparticles for drug delivery, it is seldomly utilized as a stimulus-responsive anticancer nanomedicine. Cheema *et al.* [77] reported the formation of doxorubicin (DOX)-loaded SF nanoparticles by absorption. These nanoparticles showed pH-dependent release (pH 4.5 > 6.0 > 7.4) and overcame drug resistance *in vitro*. Moreover, the SF nanoparticles had no cytotoxicity to the human breast cancer cell line MCF-7 and could be internalized and accumulated in lysosomes. These results indicated that the SF nanoparticles could be used as drug carriers for lysosomotropic delivery.

In order to achieve tumor-targeted drug delivery of DOX-loaded SF nanoparticles, the DOX-loaded magnetic SF nanoparticles were prepared using a one-step potassium phosphate salting-out strategy [78]. The introduction of superparamagnetic Fe_3_O_4_ nanoparticles not only provides magnetic tumor targeting ability for SF nanoparticles, but also realizes the artificial regulation of SF nanoparticles formation and DOX entrapment behavior. The magnetic field-induced tumor targeting ability *in vivo* and effective chemotherapy of multidrug resistant cancer demonstrates that the DOX-loaded magnetic SF nanoparticles can be utilized as a novel drug delivery system in the field of cancer therapy.

Another approach to obtain the tumor-targeted drug delivery is conjugation with tumor-specific ligand. For example, folic acid (FA), as a commonly used tumor-specific ligand for targeted delivery of anti-cancer drugs, has been conjugated on the surface of DOX-loaded SF nanoparticles [67]. Folic acid modification of SF nanoparticles not only increases the retention of the nanoparticles at the tumor site, but also promotes cellular uptake in drug delivery systems by endocytosis.

### 4.2. Protein and Growth Factor Delivery

SF nanoparticles have also been used to deliver protein and peptide drugs, especially growth factors. Growth factors are polypeptides capable of stimulating cellular growth, proliferation and differentiation, and have been widely utilized in the field of tissue regeneration [80,81,82]. However, the clinical applications of these factors often suffer from the relatively short half-lives, limited tissue penetration and potential toxicity [82]. Developing SF nanoparticles for protein and growth factor delivery is an effective way to improve therapeutic efficiency [83].

For protein delivery, the SF nanoparticles could be conjugated covalently with insulin alone with the cross-linking reagent glutaraldehyde. The recovery of the insulin-SF nanoparticles conjugates ranged from 90% to 115%. The *in vitro* half-life of the insulin-SF nanoparticles conjugates was about 2.5 times more than that of native insulin. Therefore, the SF nanoparticles have the potential values for being studied and developed as a new bioconjugate for enzyme/polypeptide drug delivery system [84]. Also, Huang *et al.* [85] fabricated fluorescein isothiocynate-labeled bovine serum albumin (FITC-BSA) loaded SF nanoparticles (FITC-BSA-SFNs) for ocular drug delivery. It was found that FITC-BSA-SFNs possessed sustained release, bioadhesive, and co-permeation characteristics. The ultrasound application significantly enhanced the penetration efficiency of FITC-BSA-SFNs as compared with passive delivery. This study suggested the combination of SF nanoparticle carriers and ultrasound may provide a novel non-invasive trans-scleral administration of macromolecular protein drugs.

For growth factor delivery, stable and negatively charged and low toxic SF nanoparticles around 150–170 nm in average diameter have been prepared via a desolvation technique using dimethyl sulfoxide as a desolvating agent [17]. These nanoparticles were accumulated in the cytosol of murine squamous cell carcinoma cells. *In vitro* release of entrapped vascular endothelial growth factor (VEGF) from SF nanoparticles showed a significantly sustained release over three weeks without initial burst, providing evidence of the potential application of nanoparticles as a growth factor delivery system. In addition, utilizing SF nanoparticles as carrier of bone morphogenetic protein-2 (BMP-2), a bone-growth regulatory factor belonging to the transforming growth factor-beta (TGF-beta) superfamily, is helpful to promote bone tissue regeneration. Shi *et al.* [86] prepared bone morphogenetic protein-2 (BMP-2)-loaded SF-based nanoparticles with a mean size of approximately 250 nm by desolvation. The BMP-2 loading efficiency is approximately 89.3%. These nanoparticles showed controlled release of BMP-2 and significantly enhanced osteogenic differentiation of mesenchymal stem cells (MSCs), which is evident in the high alkaline phosphatase (ALP) enzyme activity as well as the increased level of expression of osteogenic genes.

### 4.3. Enzyme Immobilization

Enzyme immobilization is a useful way to improve the catalytic efficiency of enzymes by increasing the half-life and stability. The SF nanoparticles possess many active amino groups and tyrosine residues and offer various possibilities for the surface modification and the covalent attachment of enzymes/drugs. Recently, SF nanoparticles have been utilized as a matrix to immobilize many enzymes, including ASNase, naringinase, neutral protease and β-glucosidase [87,88,89,90,91].

Zhang *et al.* [87] prepared SF nanoparticle-l-asparaginase conjugates using glutaraldehyde as the cross-linking reagent. The enzyme activity recovery of the immobilized l-asparaginase was about 44%. Its thermal stability clearly increased and the optimal scale of pH was much wider (pH 6~8) than that of native l-asparaginase. Using a similar method, SF nanoparticles were also conjugated covalently with naringinase or neutral protease (NP) [88,89]. Naringinase is a bienzyme that is made up of α-l-rhamnosidase and flavonoid-β-glucosidase. After eight repeated enzymatic reactions, the residual enzyme activity of the SF nanoparticles-naringinases is about 70%. For NP, after immobilization with SF nanopaticles, the stability, the optimum reactive temperature range, the optimum pH value range and the thermal stability were increased. The SF nanoparticles-naringinases and SF nanoparticles-NP bioconjugates can be utilized repeatedly by using centrifugation to separate the enzyme and the substrate. These studies indicated that the SF nanoparticles have great potential for enzyme immobilization.

In order to improve the enzyme activity recovery of the immobilized l-asparaginase (ASNase), a novel method was developed to immobilize l-asparaginase [90]. Briefly, the regenerated silk fibroin solution was mixed mildly with l-asparaginase and then was added into excess acetone. The enzyme could be embedded and immobilized in the simultaneously formed SF nanoparticles without loss of activity. The resulting SF nanoparticles-ASNase bioconjugates were crystalline globular particles of 50~120 nm in diameter with high enzyme activity recovery (90%). Moreover, the SF nanoparticles-ASNase possessed better stability in serum and greater storage stability in solution than that of native ASNase. These results indicated that SF nanoparticles are a suitable carrier to immobilize enzymes.

Using a method similar to that above, Cao *et al.* [91] prepared spherical β-Glucosidase (βG)-SF nanoparticles with a diameter of 50~150 nm. The enzyme activity recovery of βG-SF nanoparticles was 59.2%. The kinetic characteristics of the βG-SF nanoparticles are consistent with that of the free β-glucosidase. Moreover, these βG-SF nanoparticles exhibited good operational stability and could be utilized repeatedly. These results indicated that SF nanoparticles could be used as a desirable carrier for enzyme immobilization.

### 4.4. Gene Delivery

Silk fibroin-based gene delivery systems have recently been reported to provide biodegradability, biocompatibility, high transfection efficiency, and DNase resistance.

The size and sequence of the spider silk-based block copolymers that are designed via genetic engineering can be controlled [92]. Moreover, recombinant silk fibroin proteins may be further modified to gain new functions. The strategy of constructing hybrid protein at the DNA level combines the sequence encoding recombinant spider silk, responsible for the biomaterial structure, with sequences encoding the polypeptides for functionalization [93,94].

Numata K *et al.* [92] utilized genetic engineering approaches to design recombinant spider silk-based block copolymers by combining spider silk consensus repeats with DNA-binding poly(l-lysine) domains. These copolymers can interact with plasmid DNA (pDNA)-encoding GFP to assemble into pDNA complexes via ionic interactions for gene delivery. When the ratio of polymer and nucleotide is 10, the globular pDNA complexes with 30 lysine residues exhibited a particle size of 380 nm and the highest transfection efficiency (14% ± 3%). However, the transfection efficiency was too low to be used as gene carriers.

In order to enhance transfection efficiency of silk fibroin-based gene carriers, arginine-glycine-aspartic acid (RGD) cell-binding domain and DNA-binding poly(l-lysine) domains were incorporated with spider silk consensus repeats by genetic engineering to successfully fabricate recombinant spider silk-polylysine-RGD block copolymers for gene delivery [95]. When the ratio of numbers of amines to phosphates of DNA (N/P) is 2, the resulting globular pDNA complexes with 30-lysine residues and 11 RGD sequences exhibited a particle size of 168 nm and showed the highest transfection efficiency because of integrin-mediated transfection with 11 RGD sequences. This study suggested that the globular pDNA complexes of spider silk-polylysine-RGD block copolymers have potential application in the field of gene delivery.

The silk fibroin-based gene carriers could also be functionalized with cell penetrating and cell membrane destabilizing peptides such as ppTG1 peptide to enhance transfection efficiency. The ppTG1 peptide destabilized the cell membrane and enhanced gene transfection efficiency. Moreover, the transfection efficiency of the globular pDNA complexes of recombinant spider silk-polylysine-ppTG1 dimer with a particle size of 99 nm was comparable to that of the transfection reagent Lipofectamine 2000 [96].

In order to achieve the objective of tumor targeting gene delivery, recombinant spider silk-based nanoparticles containing DNA-binding domains poly(l-lysine) and tumor-homing peptides (THPs) such as F3 (KDEPQRRSARLSAKPAPPKPEPKPKKAPAKK), Lyp1 (CGNKRTRGC), and CGKRK have been developed to deliver target-specific plasmid DNA (pDNA) to the tumor cells (MDA-MB-435 and MDA-MB-231) with low cytotoxicity, significant enhancement of target specificity to tumor cells and high efficiency [97,98]. The pDNA complexes of the recombinant spider silk proteins containing poly(l-lysine) and tumor-homing peptides (THPs) exhibit a globular morphology and particle size from about 150 to 250 nm. By introducing F3 and CGKRK THPs, the tumor targeting ability of the pDNA complexes is improved significantly [97]. Similarly, by additions of F3 and Lyp1, the globular pDNA complexes with about 100 nm in diameter of recombinant silk proteins also suggest enhanced tumor targeting ability [98].

## 5. Conclusions and Future Perspectives

Owing to their good biocompatibility, degradability, and nontoxicity, silk fibroin (SF) nanoparticles have been investigated as a promising carrier for delivery of various drugs, including small molecule drugs, protein drugs, and gene drugs, *etc.* Absorption and bioavailability of drugs encapsulated into SF nanoparticles can be improved. The aqueous processability of SF allows nanoparticle formation under mild conditions. The biodegradation rate of SF can be regulated by changing its degree of crystallinity, molecular weight or degree of crosslinking [99,100,101]. In addition to these useful properties, SF possesses several active amino groups and tyrosine residues and can be easily modified to add new functions.

Despite the excellent properties of SF nanoparticles that make it a promising drug carrier, it is still necessary to address some critical challenges. Each nanoparticle preparation method has pros and cons, and it is important to continue to develop novel nanoparticles fabrication techniques to match different demands. Also, the predictions for degradation of SF and drug release kinetics of SF-based nanoparticle drug delivery systems is difficult owing to the variation of SF between both species and individuals of the same species. Using genetically recombinant SF-based nanoparticles for drug delivery as alternatives to native ones may overcome such deficiencies [102]. Moreover, the drug delivery systems may exhibit low therapeutic efficiency and toxic problems due to lack of specificity and functionality. The surface engineering strategies including genetic engineering or surface chemical modification can be developed to improve the therapeutic efficiency. With the development of technology, silk fibroin-based nanoparticles have potential for a wider application.
